# Use of very short answer questions compared to multiple choice questions in undergraduate medical students: An external validation study

**DOI:** 10.1371/journal.pone.0288558

**Published:** 2023-07-14

**Authors:** Elise V. van Wijk, Roemer J. Janse, Bastian N. Ruijter, Jos H. T. Rohling, Jolein van der Kraan, Stijn Crobach, Mario de Jonge, Arnout Jan de Beaufort, Friedo W. Dekker, Alexandra M. J. Langers

**Affiliations:** 1 Center for Innovation in Medical Education, Leiden University Medical Center, Leiden, The Netherlands; 2 Department of Clinical Epidemiology, Leiden University Medical Center, Leiden, The Netherlands; 3 Department of Gastroenterology and Hepatology, Leiden University Medical Center, Leiden, The Netherlands; 4 Department of Cell and Chemical Biology, Leiden University Medical Center, Leiden, The Netherlands; 5 Department of Pathology, Leiden University Medical Center, Leiden, The Netherlands; 6 Leiden University Graduate School of Teaching, Leiden University, Leiden, The Netherlands; Ankara University Faculty of Medicine: Ankara Universitesi Tip Fakultesi, TURKEY

## Abstract

Multiple choice questions (MCQs) offer high reliability and easy machine-marking, but allow for cueing and stimulate recognition-based learning. Very short answer questions (VSAQs), which are open-ended questions requiring a very short answer, may circumvent these limitations. Although VSAQ use in medical assessment increases, almost all research on reliability and validity of VSAQs in medical education has been performed by a single research group with extensive experience in the development of VSAQs. Therefore, we aimed to validate previous findings about VSAQ reliability, discrimination, and acceptability in undergraduate medical students and teachers with limited experience in VSAQs development. To validate the results presented in previous studies, we partially replicated a previous study and extended results on student experiences. Dutch undergraduate medical students (n = 375) were randomized to VSAQs first and MCQs second or vice versa in a formative exam in two courses, to determine reliability, discrimination, and cueing. Acceptability for teachers (i.e., VSAQ review time) was determined in the summative exam. Reliability (Cronbach’s α) was 0.74 for VSAQs and 0.57 for MCQs in one course. In the other course, Cronbach’s α was 0.87 for VSAQs and 0.83 for MCQs. Discrimination (average R_ir_) was 0.27 vs. 0.17 and 0.43 vs. 0.39 for VSAQs vs. MCQs, respectively. Reviewing time of one VSAQ for the entire student cohort was ±2 minutes on average. Positive cueing occurred more in MCQs than in VSAQs (20% vs. 4% and 20.8% vs. 8.3% of questions per person in both courses). This study validates the positive results regarding VSAQs reliability, discrimination, and acceptability in undergraduate medical students. Furthermore, we demonstrate that VSAQ use is reliable among teachers with limited experience in writing and marking VSAQs. The short learning curve for teachers, favourable marking time and applicability regardless of the topic suggest that VSAQs might also be valuable beyond medical assessment.

## Introduction

Assessment in the educational field commonly uses Multiple Choice Questions (MCQs), because this question type offers high reliability and easy machine-marking. However, it also allows for cueing (i.e., answering questions based on cues in the question or answer options rather than on content knowledge) and stimulates a recognition-based study approach [1–4]. Although recognition may be sufficient to pass a MCQ-based assessment, oftentimes MCQs are not representative for a future situation in which the assessed knowledge has to be applied, for instance because of the absence of a demarcated set of possible answers. This is, among others, the case in medical education, where it has been critically noted that clinical practice does not offer a multiple choice list of possible diagnoses or procedures, nor is there a single best recognisable answer in the medical profession [5, 6].

Although other question formats have been proposed to circumvent the limitations of MCQs, such as uncued questions and extended matching questions [6–8], these question formats may still facilitate a recognition-based study approach. Very Short Answer Questions (VSAQs), a free-response type of questions with the answer being limited to 1–4 words, may be better suited to circumvent some of the general limitations of MCQs. The open-ended nature of the VSAQs may prevent surface-level study approaches and cueing [4, 9–11], and it may better represent a profession’s real-life practice, such as the medical profession, where VSAQs better reflect clinical practice. In addition, VSAQs are better able to discriminate between students based on proficiency in the content knowledge [9–14] and may increase retention of knowledge [15–17].

Although the use of VSAQs in medical assessments is increasing, evidence regarding validity and reliability of this question type in the medical setting is mainly based on studies from a single research group, consisting of teachers experienced in developing and marking VSAQs [4, 10, 13, 14, 18]. In one study, Sam *et al*. (2018) [13] compared 3^rd^ year medical students starting a test with either VSAQs or MCQs, followed by questions in the opposite format. They observed a higher reliability (Cronbach’s α: 0.91 vs. 0.85) and lower mean test score (52.4% vs. 69.7%) for VSAQs vs. MCQs, respectively. Moreover, two-third of students (strongly) agreed that VSAQs better represented clinical practice and half of the students (strongly) agreed VSAQs better prepared them for clinical practice. Cueing was more strongly associated with MCQs. In 5^th^ year pathology students, higher reliability (Cronbach’s α: 0.86 vs. 0.76) and a lower median test score (72% vs. 80%) were found in VSAQs vs. MCQs [14]. Lastly, across 20 UK medical schools, Sam and colleagues [18] found a 21% higher test score for MCQs compared to VSAQs, as well as a higher positive cuing rate in MCQs. In this study, they reported marking VSAQs to be feasible.

However, it remains unclear whether the application of VSAQs by teachers with less experience in writing and marking VSAQs, in a different population, country, and medical educational setting yields the same results. Before VSAQs can be implemented in a wider context, more evidence is needed. Therefore, we aimed to externally validate the positive results of VSAQs regarding reliability, discrimination, and acceptability in a cohort of Dutch medical undergraduate students with non-expert teachers. Additionally, we wanted to explore the impact of VSAQs on cueing effects and student experiences of VSAQ-assessment. In order to achieve these aims, we partially replicated the study design of Sam *et al*.[13].

## Methods

### Setting

**Fig 1:**
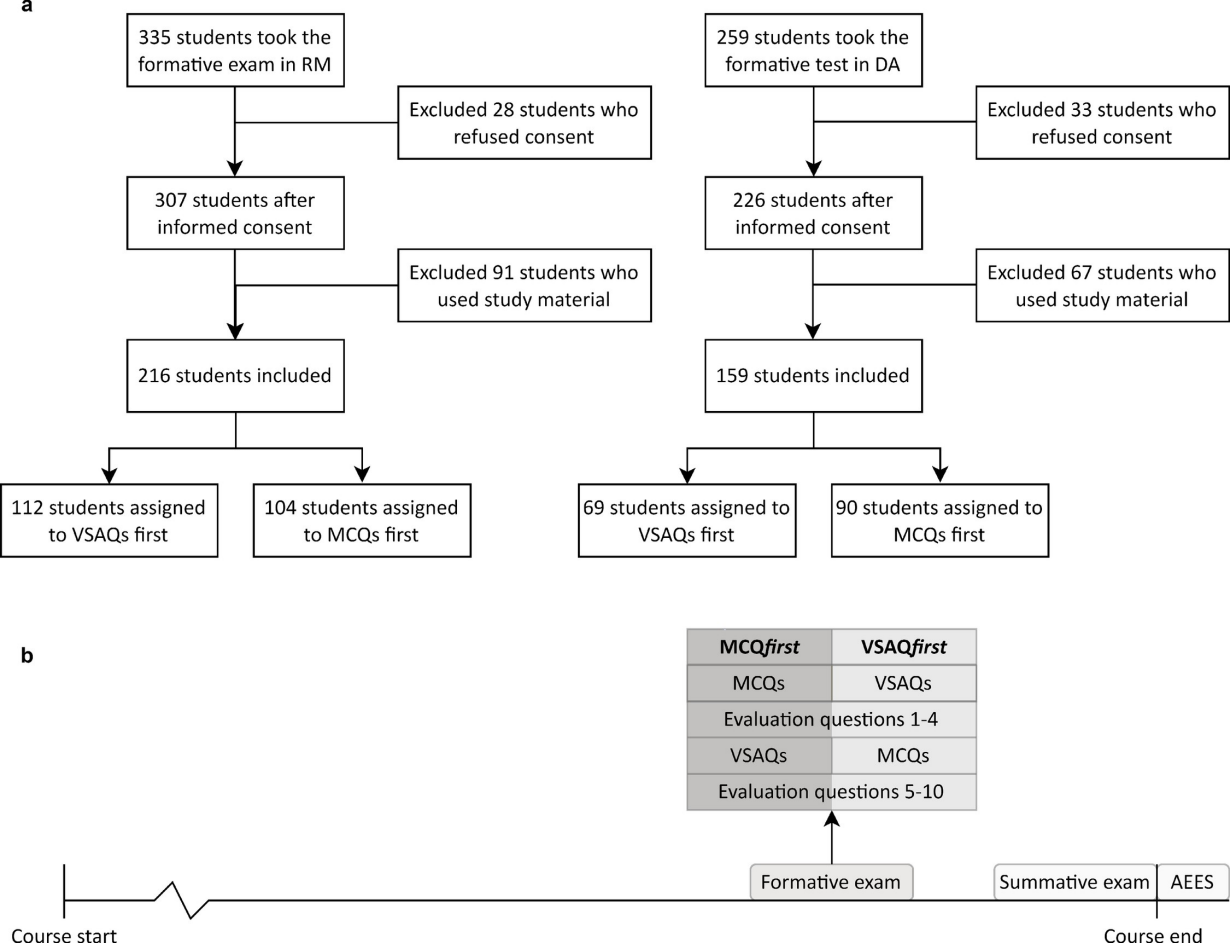
(A) Set-up of both courses (RM and DA) with the formative exam and contents, summative exam and contents, and the Automated Education Evaluation System (AEES) (B) Flowchart of the study participants.

This study was simultaneously performed in two different student cohorts (cohort 2019 and cohort 2020) using the same study design. First year students (cohort 2020) followed the fundamental course “*Regulation and Metabolism*” (RM, May 2021) and the second year students (cohort 2019) followed the clinical course *“Diseases of the Abdomen”* (DA, April 2021) in the bachelor of Medicine at the Leiden University Medical Center (LUMC), the Netherlands. Both courses (6 and 7 weeks, respectively) cover metabolic and gastrointestinal topics. During the course, students had weekly mini-exams in DA where they were presented 2–3 VSAQs, but not in RM. Near the end of these courses, students are offered a formative exam and the courses end with a summative exam. After the summative exam, students can evaluate the course with the Automated Education Evaluation System (AEES, **Fig 1A**), which includes questions on constructive alignment. Relevant AEES questions for this study were answered using a 5-point Likert-scale (strongly disagree, disagree, neutral, agree, strongly agree). The LUMC uses RemindoToets (Paragin) [19] for digital assessment with the possibility of proctoring. The current study included the formative and summative exams in both courses for analyses.

### Formative exam

To determine reliability, discrimination, cueing effects, and students’ insights in the formative exams of the RM and DA courses, students were randomly assigned to a group starting with MCQs (RM-MCQ*first* and DA-MCQ*first*) or starting with VSAQs (RM-VSAQ*first* and DA-VSAQ*first*), followed by identical questions in the opposing format, similar to the study of Sam *et al*. [13] (**Fig 1A**). When a section (i.e. either the VSAQ part or the MCQ part) of the exam was finished, students were not able to revisit this specific section. For instance, a student starting the exam with VSAQs could not go back to the VSAQs nor change their answer when they started the section with MCQs. However, it was possible to revisit items or change the response within the same section of the exam before closing the section. The topics in the formative assessment covered the entire spectrum of the course. The MCQs used in the formative exam were written by the course directors with the intent to test the course learning goals. The VSAQs were written in the same way, with assistance from the research team (RJJ, AMJL) to create VSAQs of good quality. The research team was familiar with the literature on VSAQs, but did not yet have any experience in writing VSAQs. For DA, new questions were created as open ended questions suitable for the VSAQ format, and then four answer options for each question were generated to create the parallel MCQ. In RM, existing MCQs that passed the cover test (i.e. the answer can be given without reading the answer options) were transformed into VSAQs by removing the answer options. If the existing MCQs were not specific enough, adjustments to the questions were made to fulfil the VSAQ requirements. Thus, for DA, 24 completely identical questions in both formats were asked, while for RM, 25 questions that tested the same knowledge were asked, albeit sometimes worded differently. The formative exam was available in a fixed timeslot. Participation in this formative exam was mandatory in RM and optional in DA. The exam format order per student was determined using a random number generator in Microsoft Excel (i.e., a Mersenne Twister algorithm) [20]. Only students who gave informed consent were included in the analysis.

After having finished the first part of the formative exam (either MCQs or VSAQs) students were asked to rate three statements on a 5-point Likert scale (strongly disagree, disagree, neutral, agree, strongly agree) and one question ranging from 1 to 10, based on the specific question format with which they were just tested: 1) *The questions are a good representation of how I would be expected to answer questions in clinical practice;* 2) *I found the questions easy;* 3) *I was often unsure whether my answer would be correct)*; 4) *If I had to give an estimate of the grade I would have achieved based on these questions*, *my estimate would be <grade>*. Because these statements were presented to the students after they finished the first part of the exam, half of the students answered the questions after having finished MCQs only and half of the students after having finished VSAQs only. After the students answered the four evaluation questions, they continued with the second part of the formative test, in which they had to answer the exam-questions in the opposite format. At the end of the second part of the formative exam, all students were asked to rate six more general statements about both question formats: 5) *VSAQs are easier than MCQs*; 6) *VSAQs are more in line with daily clinical practice than MCQs;* 7) *I prepare differently for an assessment with VSAQs than for an assessment with MCQs;* 8) *VSAQs would be a better preparation for clinical practice than MCQs*; 9) *Through the use of VSAQs*, *the test is better aligned with this course*, *than a test using MCQs;* and 10) *Any comments I would like to add*: *<open question>*. Finally, for research purposes, students were asked whether they used during the formative exam. Given that there were no negative repercussions to using study materials and this was clear to students, we believe that the answer to this question reflects the actual use of study material in the majority of students. Students who used study materials were excluded from the analysis.

We determined reliability and discriminative capability for content knowledge. The average score, calculated over MCQs and VSAQs separately, was stratified by whether students took MCQs or VSAQs first. Cueing was measured by comparing the answer to an MCQ with the answer to the corresponding VSAQ. We looked at cueing per question (i.e., how often did cueing occur per individual question) and cueing per person (i.e., in how many questions did cueing occur per individual student). We discerned positive and negative cueing. In positive cueing, students used clues in either the MCQ question and/or answer options to arrive at the right answer, which was not possible in VSAQs because no answer options were available. In our study, this could be observed when a student answered a VSAQ incorrectly, but the equivalent MCQ correctly. Negative cueing happens when students are misled by an incorrect answer option in a MCQ (e.g., due to a distractor that is too plausible). In our study, this was derived from a student being able to answer the VSAQ correctly, but not able to give the correct answer to the equivalent MCQ. Although it might have been of influence, the probability of guessing the right answer could not be taken into account. Students’ insights were determined from the evaluation questions asked midway through and at the end of the formative exam.

### Summative exam

The summative exams of RM and DA were rewritten to replace part of the MCQs with VSAQs (45 in RM and 16 in DA). For RM, this was done through rewriting existing MCQs, whereas for DA, a 2-hour workshop was organized for teachers on how to write VSAQs. Question writers in both courses received written instructions about how to write VSAQs based on information provided by the author of the initial paper on VSAQs, as can currently be found in the publication by Bala *et al*. [13, 21]. For each learning goal, questions were initially written by the experts with content knowledge (e.g., gynaecology questions were written by a gynaecologist). The research team (RJJ, AMJL) assisted, if necessary, in adjusting the questions to the optimal VSAQ format. At the end of both exams, preapproved answers to the VSAQs were automatically marked as correct. Subsequently, teachers reviewed all incorrect answers and could easily add answers that were not in the predefined list, but were also found to be correct. The grading was done by one teacher; a second teacher was consulted when there were doubts about certain answers.

VSAQ review time per question for each teacher was recorded in DA to determine acceptability. The total reviewing time per question was recorded by the reviewer using the timer function on a smartphone. Reviewing time started when the reviewer first looked at the question and ended when the question was fully resolved. This included both reviewing the answers and discussion with other teachers when necessary. Because the marking of only a few VSAQs of the exam was recorded during the initial data collection, which impeded a correct and unselected overview of the reviewing time, one year later the reviewing time of all VSAQs in that year’s summative exam was collected again. The AEES questionnaire was supplemented with two questions regarding students’ insights: 1) *Because I knew that I would be tested by very short answer questions*, *I studied in another way than I normally would*; and 2) *Through the use of very short answer questions*, *the test was a better representation of what I learned in this course*, *compared to a test using multiple-choice questions*. In addition, the perceived alignment between teaching and assessment was compared to the alignment of the course in the years 2017, 2018, and 2019, determined from two pre-existent questions in the AEES questionnaire: 1) *The assessment as a whole (form and content) is appropriate for what you should have mastered at the end of the course*; and 2) *The (online) test formats (e*.*g*. *MCQs*, *open questions*, *oral and written presentations*, *practical assessments) matched what I have learned*. Due to emergency remote teaching during COVID-19, 2020 is not considered in these comparisons.

### Statistical analysis

Continuous variables are presented as mean (standard deviation) or median (interquartile range) depending on their distribution. Categorical variables are presented as number (proportion). Reliability was determined by calculating the Cronbach’s α or the VSAQs and MCQs in both formative exam formats, which is a measure of internal correlation between items on a test level [22, 23]. A higher Cronbach’s α indicates better reliability with values of 0.7 or higher indicating acceptable reliability. The discriminative capability for content knowledge was determined using the mean of the R_ir_-values of each question, where the R_ir_-value is the correlation between one test-item and other test-items [24]. Items with a R_ir_-value of more than 0.25 typically represent items with an adequate discriminative capability. Mean test scores were calculated as the percentage of correctly answered questions. Reviewing time was expressed in minutes and seconds. All statistical analyses were performed using R version 4.1.0 (R Foundation for Statistical Computing, Vienna, Austria).

### Ethical approval

This study was reviewed and approved by the Educational Research Review Board of the Leiden University Medical Center (file number: OEC/ERRB/20201208/1).

## Results

Of the 335 students who took the formative exam in RM, 216 students were included in our study. In DA, 159 of the 259 students who took the formative exam were included (**Fig 1B**). In RM, 104 students started with MCQs (RM-MCQ*first*) and 112 students started with VSAQs (RM-VSAQ*first)*. In DA, 90 students were assigned to DA-MCQ*first* and 69 students to DA-VSAQ*first* The summative exam was made by 352 students in RM and 308 students in DA.

### Reliability and discrimination

We compared the VSAQs of students starting with VSAQs with the MCQs of students starting with MCQs. This comparison reflects the results of the VSAQs and MCQs that are not influenced by prior questions. VSAQs had higher reliability compared to MCQs (Cronbach’s α 0.74 vs. 0.57 in RM; 0.87 vs. 0.83 in DA for VSAQs vs. MCQs, respectively) (**Table 1**). In the same students, discrimination (mean [SD]), expressed as the R_ir_-value, was higher in VSAQs compared to MCQs (0.27 [0.15] vs. 0.17 [0.13] in RM; 0.43 [0.10] vs. 0.39 [0.10] in DA, for VSAQs vs. MCQs, respectively). The mean scores (mean [SD]) were lower and had a wider distribution width for VSAQs compared to MCQs (57.0 [15.7] vs. 71.2 [12.2] in RM; 51.6 [23.9] vs. 70.0 [19.7] in DA, for VSAQs vs. MCQs, respectively). These results were similar when comparing results within groups (e.g., VSAQs vs. MCQs within MCQ*first*).

**Table 1:** Cronbach’s alpha, average R_ir_ score and mean (SD) scores for the MCQs and VSAQs in MCQ*first* and VSAQ*first*.

	Regulation and Metabolism				Diseases of the Abdomen			
	MCQ*first*		VSAQ*first*		MCQ*first*		VSAQ*first*	
	MCQ (*n* = 104)	VSAQ (*n* = 104)	VSAQ (*n =* 112)	MCQ (*n* = 112)	MCQ (*n* = 90)	VSAQ (*n* = 85)	VSAQ (*n* = 69)	MCQ (*n* = 64)
Cronbach’s α	0.57	0.61	0.74	0.71	0.83	0.90	0.87	0.71
Average R_ir_ (SD)	0.17 (0.13)	0.19 (0.14)	0.27 (0.15)	0.27 (0.09)	0.39 (0.10)	0.49 (0.13)	0.43 (0.10)	0.26 (0.10)
Mean score (SD), %	71.2 (12.2)	72.3 (12.9)	57.0 (15.7)	75.4 (14.1)	70.0 (19.7)	58.4 (25.9)	51.6 (23.9)	72.5 (15.0)

MCQ, multiple choice question; VSAQ, very short answer question; SD, standard deviation.

### Acceptability

In the initially collected data, the average reviewing time per VSAQ by one teacher in the summative exam of DA (7 VSAQs, 308 students) was 2 minutes and 20 seconds (SD 52 seconds). Additionally, on average 2 minutes and 9 seconds (SD 2 minutes and 36 seconds) were spent replying to comments and consultation of other teachers. The maximum time spent on a single VSAQ was 11 minutes and 24 seconds. One year later (22 VSAQs, 338 students), the average time spent on reviewing questions in DA was 1 minute and 58 seconds (SD 40 seconds) and consultation of other teachers took on average 36 seconds (SD 47 seconds).

### Secondary outcomes

Positive cueing, defined as a correctly answered MCQ with an incorrectly answered equivalent VSAQ, occurred on average more often per student in RM-VSAQ*first* and DA-VSAQ*first* (20.0%, IQR; 16.0–28.0%; 20.8%, IQR; 12.5–29.2%, respectively) compared to RM-MCQ*first* and DA-MCQ*first* (4.0%, IQR; 4.0–8.0%; 8.3%, IQR; 4.2–16.7%, respectively) (**Table 2**). On a question level, positive cueing occurred in 100% of questions in all groups. The frequency of positive cueing per question was on average higher in RM-VSAQ*first* and DA-VSAQ*first* (14.3%, IQR 7.1–33.9%; 22.7%, IQR 10.9–28.5%, respectively) compared to RM-MCQ*first* and DA-MCQ*first* (4.8%, IQR 2.9–9.6%; 15.9%, IQR 11.8–20.3%, respectively) (**Table 3**). Negative cueing in students, which was defined as students answering the VSAQ correctly and the equivalent MCQ incorrectly, occurred more often per student in RM-MCQ*first* compared to RM-VSAQ*first* (8.0%, IQR 4.0–12.0% to 4.0%, IQR 0.0–4.0%). In DA-MCQ*first*, negative cueing was on average not observed in students (0.0%, IQR 0.0–4.2%). Negative cueing per question occurred in 92%, 56%, 79%, and 79% of the questions for RM-MCQ*first*, RM-VSAQ*first*, DA-MCQ*first*, and DA-VSAQ*first*, respectively. The frequency of negative cueing per question was on average lower in RM-VSAQ*first* compared to RM-MCQ*first* (0.9%, IQR 0.0–1.8%; 3.8%, IQR 1.9–12.5%), but higher in DA-VSAQ*first* compared to DA-MCQ*first* (3.1%, IQR 1.6–5.1%; 1.8%, IQR 1.2–3.5%). The maximum percentage of positive cueing by students in a single question was the highest in RM-MCQ*first* (62.5%). The maximum percentage of negative cueing by students in a single question was 38.5% in RM-MCQ*first*.

**Table 2:** Positive and negative cueing per person in MCQ*first* and VSAQ*first*.

	Regulation and Metabolism		Diseases of the Abdomen	
	MCQ*first* (*n =* 104)	VSAQ*first* (*n =* 112)	MCQ*first* (*n =* 90)	VSAQ*first* (*n =* 69)
Positive cueing, median (IQR), %	4.0 (4.0–8.0)	20.0 (16.0–28.0)	8.3 (4.2–16.7)	20.8 (12.5–29.2)
Negative cueing, median (IQR), %	8.0 (4.0–12.0)	4.0 (0.0–4.0)	0.0 (0.0–4.2)	4.2 (0.0–4.2)

MCQ, multiple choice question; VSAQ, very short answer question; IQR, interquartile range.

**Table 3:** Positive and negative cueing per question in MCQ*first* and VSAQ*first*.

	Regulation & Metabolism		Diseases of the Abdomen	
	MCQ*first* (*n* = 104)	VSAQ*first* (*n* = 112)	MCQ*first* (*n* = 90)	VSAQ*first* (*n* = 69)
**Frequency of questions where cueing occurred, %**				
Positive cueing	100	100	100	100
Negative cueing	92	56	79	79
**Average frequency of cueing per question, %**				
Positive cueing, median (IQR)	4.8 (2.9–9.6)	14.3 (7.1–33.9)	15.9 (11.8–20.3)	22.7 (10.9–28.5)
Positive cueing, max	26.9	62.5	32.9	43.8
Negative cueing, median (IQR)	3.8 (1.9–12.5)	0.9 (0.0–1.8)	1.8 (1.2–3.5)	3.1 (1.6–5.1)
Negative cueing, max	38.5	16.1	7.1	10.9

MCQ, multiple choice question; VSAQ, very short answer question; IQR, interquartile range.

**Fig 2:**
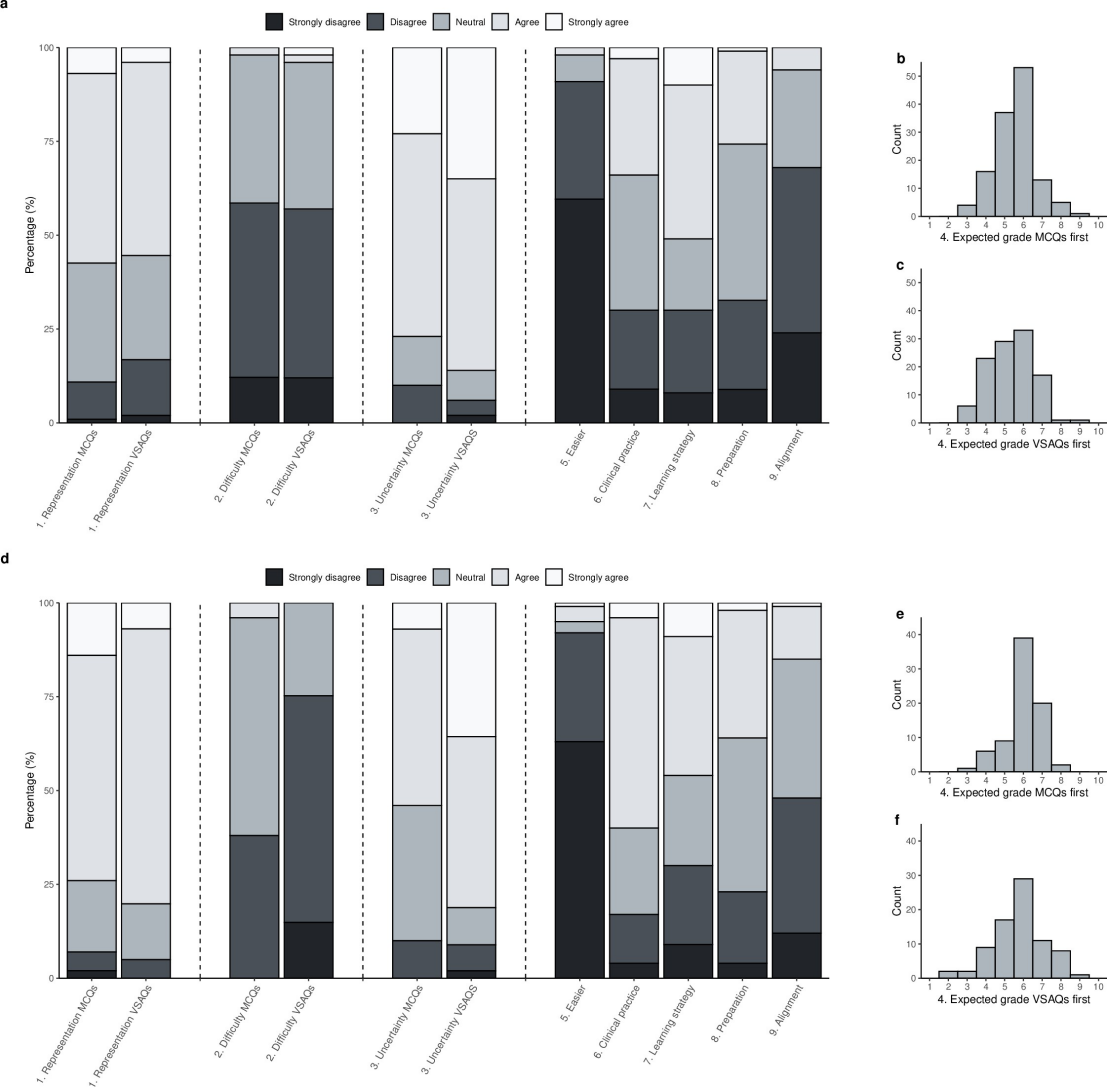
Students’ experiences and grade estimates of the MCQs and VSAQs in the formative exam. Distribution of the answers given to the 5-point Likert scale evaluation questions halfway through the exam after the MCQs or VSAQs and at the end of the exam; and estimates of their grade halfway through the exam in RM (A, B, C) and DA (D, E, F).

When asked whether they found the questions easy, students who had been answering only VSAQs more often disagreed compared to students who had been answering MCQs only in the DA course (EQ2: 3, IQR 2–3 vs. 2, IQR 2–2), but students estimated their final grade to be higher if they had started with VSAQs (**S1 Table**). More than 80% of students were uncertain about answering VSAQs correctly (86% and 82% in RM and DA, respectively) (**S2 Table and Fig 2**). Also at the end of the formative exam, after having answered questions in both formats, approximately 90% of students (strongly) disagreed that VSAQs were easier than MCQs (**S3 Table**). 51% of students in RM and 46% in DA (strongly) agreed that assessment with VSAQs changed their test preparation. In DA, 60% of students agreed or strongly agreed that VSAQs better represented clinical practice. This was 34% in RM. Almost 70% of students in RM and 48% in DA (strongly) disagreed that the test was better aligned with the course by using VSAQs. 45% of students in RM and 42% in DA (strongly) agreed they would change learning behavior if tested with VSAQs (**S4 Table**). 83% of students in RM (strongly) disagreed with the statement that the use of VSAQs made the exam a better representation of what they learned during the course compared to MCQs. This was 51% in DA. Perceived alignment of assessment, teaching and learning activities are reported in **S5 Table**.

## Discussion

In this study we aimed to externally validate the earlier results regarding reliability, discrimination, and acceptability of VSAQs compared to MCQs in a cohort of Dutch medical undergraduate students, based on earlier work by Sam *et al*. [13]. In accordance with their findings, we observed higher reliability and discrimination of VSAQs compared to MCQs, with an acceptable time to mark VSAQs. Results were more positive in DA than in RM, which might be attributable to the workshop offered to the teachers, better suitable course material, and the opportunity for students to practice with the VSAQs prior to the exams. Additionally, we explored the impact of VSAQs on cueing effects, perceived alignment between assessment and teaching, and student experiences of VSAQs. Cueing effects occurred less frequently in VSAQs compared to MCQs. Students noted a high level of uncertainty when answering VSAQs and around half of students prepared differently for VSAQs. More than half of the students thought VSAQs better represented clinical practice. However, perceived constructive alignment seemed to diminish in RM and not improve in DA.

The higher reliability and discrimination but lower test scores of VSAQs compared to MCQs may in part reflect the decreased possibility of guessing correctly in VSAQs, and are line with Sam *et al*. [13] and other previous studies [14, 18]. The lower score also suggests that VSAQs are more difficult, possibly due to a need of answer generation, rather than answer recognition, which provides a better measure of a students’ true content knowledge and increases validity [4, 13, 14]. The high discriminative capability of VSAQs is further supported by higher average R_ir_ values of VSAQs in DA. In RM, average R_ir_ values were relatively low for both MCQs and VSAQs, although an increase in R_ir_ value in VSAQs compared to MCQs could still be observed.

The teachers who graded the VSAQs deemed the reviewing time of VSAQs acceptable. This is supported by previous studies that found comparable and shorter review times, using different marking systems, multiple examiners, and more questions [13, 14]. Nonetheless, whereas not every MCQ has to be reviewed, it should be noted that a VSAQ should always be reviewed after machine marking, although repeated use of questions may decrease reviewing time, depending on software used [13].

Positive cueing per student occurred more often in the students who started with VSAQs, which is in line with the findings of Sam *et al*. [13]. This is expected, as students answering the VSAQs first and MCQs second cannot carry over the MCQ answer to the VSAQ, therefore having to rely on content knowledge for the VSAQ. Cueing per question was also seen more often in this group, but not for every question [13]. However, we most likely also measured students guessing the right answer, as it is nearly impossible to separate guessing and cueing in MCQs [11]. Negative cueing differed only slightly between groups, similar to Sam *et al*. who observed similar negative cueing between groups [13]. It should be noted that in many questions cueing occurred, but per question cueing was observed in few students.

Looking at students’ experiences, we found results comparable with Sam *et al*. [13]. The vast majority of the students thought the VSAQs were more difficult than MCQs and almost half of the students said they changed their learning behavior because they were assessed with VSAQs. We observed several noteworthy differences in student experiences between courses that may serve as primer for future research. Concerning clinical practice, students of DA were more positive than students of RM, possibly due to the clinical content in DA having been a better fit for VSAQs than the more fundamental content of RM. This indicates the importance of identifying areas that will benefit most from assessment with VSAQs [21]. Feedback provided by students mainly indicated that VSAQ phrasing might not always have been clear enough. This led to uncertainty regarding the level of specificity of the desired answer, highlighting the importance of a well-designed VSAQ with specific lead-ins [4, 21, 25]. Additionally, a majority of students in RM considered VSAQs to be a poorer representation of course content, while this was only half of the students in DA. This may in part be due to the differences in course content, but uncertainty as a result of an unclearly formulated question may also have played a role. The student feedback in RM possibly also reflects insufficient preparation for the new question format during the course, as students in DA were exposed to VSAQs at multiple timepoints throughout the course. If students have more time to practice, their ability to answer VSAQs may improve [21].

Study strengths are the randomized design, studying two different courses, and the investigation of student perspectives. Furthermore, the fact that teachers who participated in our study had limited experience with VSAQs allowed us to validate the previous results in an independent setting with less experienced teachers. Limitations are the seemingly poor question quality in the formative RM exam, and the relatively small sample size. Furthermore, due to the low-stakes nature of the formative exam, we cannot be certain that students performed at their best when answering the questions. To determine acceptability, we used only one reviewer who logged the times by hand, leading to less accurate reviewing times. To obtain a more precise measure of acceptability, these findings could be extended by using multiple examiners, more VSAQs and automatically logged times.

Although we validated the VSAQs and investigated student experiences in a medical cohort, we believe that the strengths of VSAQs compared to MCQs are generalizable to other educational fields. Especially, student experiences were mainly related to VSAQs without a focus on a medical context. Real life situations rarely offer a clear single best answer or a list of possible answers. Moreover, in any field open essay questions or other higher-order questions are costly to implement. Although further studies should extend these results to general higher education, our results show VSAQs may provide a promising alternative to MCQ-based assessment in education in general.

In conclusion, this study confirms the positive results of Sam *et al*. [13] on VSAQs in terms of reliability, discrimination, and acceptability in formative assessments in a Dutch cohort of undergraduate medical students. Additionally, these results were confirmed in teachers with only limited prior VSAQ experience and previous results on student experiences are extended. Wider implementation of VSAQs in medical education seems justified and may also improve assessment in other fields of higher education.

## Supporting information

S1 Table Median (IQR) scores of the 5-point Likert scale evaluation questions (EQ1-3 and EQ5-9) and estimated grade question (EQ4) in the formative exam. EQ1-4 halfway of the exam after the MCQs (MCQ*first*) or VSAQs (VSAQ*first*); EQ5-EQ9 at the end of the exam after both MCQs and VSAQs.
(DOCX)

S2 Table Distribution of the answers given to the 5-point Likert scale evaluation questions halfway of the formative exam after MCQs (MCQ*first*) or VSAQs (VSAQ*first*). (DOCX)

S3 Table Distribution of the answers given to the 5-point Likert scale evaluation questions at the end of the formative exam. (DOCX)

S4 Table Median (IQR) scores and distribution of the answers given to the 5-point Likert scale evaluation questions after the summative exam. (DOCX)

S5 Table Median (IQR) scores of the 5-point Likert scale questions on constructive alignment after the summative exam (1: strongly disagree, 2: disagree, 3: neutral, 4: agree, 5: strongly agree). (DOCX)
