# Supplementary material for: Use of very short answer questions compared to multiple choice questions in undergraduate medical students: An external validation study
Source: PLoS One. 2023 Jul 14;18(7):e0288558. doi: 10.1371/journal.pone.0288558 (PMC10348524; doi:10.1371/journal.pone.0288558)
Supplement: S1 Table — EQ1-4 halfway of the exam after the MCQs (MCQfirst) or VSAQs (VSAQfirst); EQ5-EQ9 at the end of the exam after both MCQs and VSAQs. (DOCX) [file pone.0288558.s001.docx]

## **S1 Table**. **Median (IQR) scores of the 5-point Likert scale evaluation questions (EQ1-3 and EQ5-9) and estimated grade question (EQ4) in the formative exam. EQ1-4 halfway of the exam after the MCQs (MCQfirst) or VSAQs (VSAQfirst); EQ5-EQ9 at the end of the exam after both MCQs and VSAQs.**

|  | **Regulation and Metabolism** | | **Diseases of the Abdomen** | |
| --- | --- | --- | --- | --- |
|  | **MCQ*first*  (*n* = 104)** | **VSAQ*first*  (*n* = 112)** | **MCQ*first*  (*n* = 85)** | **VSAQ*first*  (*n* = 64)** |
| EQ1 | 4 (3-4) | 4 (3-4) | 4 (3-4) | 4 (4-4) |
| EQ2 | 2 (2-3) | 2 (2-3) | 3 (2-3) | 2 (2-2) |
| EQ3 | 4 (4-4) | 4 (4-5) | 4 (3-4) | 4 (4-5) |
| EQ4 | 6 (5-6) | 6 (6-7) | 5 (4-6) | 6 (5-6) |
| EQ5 | 1 (1-2) | | 1 (1-2) | |
| EQ6 | 3 (2-4) | | 4 (3-4) | |
| EQ7 | 4 (2-4) | | 3 (2-4) | |
| EQ8 | 3 (2-4) | | 3 (2-4) | |
| EQ9 | 2 (2-3) | | 2 (2-3) | |

MCQ, multiple choice question; VSAQ, very short answer question.

EQ1: *The questions are a good representation of how I would be expected to answer questions in clinical practice.*

EQ2: *I found the questions easy.*

EQ3: *I was often unsure whether my answer would be correct.*

EQ4: *If I had to give an estimate of the grade I would have achieved based on these questions, my estimate would be <grade>.*

EQ5: *VSAQs are easier than MCQs*.

EQ6: *VSAQs are more in line with daily clinical practice than MCQs.*

EQ7: *I prepare differently for an assessment with VSAQs than for an assessment with MCQs.*

EQ8: *VSAQs would be a better preparation for clinical practice than MCQs*.

EQ9: *Through the use of VSAQs, the test is better aligned with this course, than a test using MCQs.*
