# Supplementary material for: Use of very short answer questions compared to multiple choice questions in undergraduate medical students: An external validation study
Source: PLoS One. 2023 Jul 14;18(7):e0288558. doi: 10.1371/journal.pone.0288558 (PMC10348524; doi:10.1371/journal.pone.0288558)
Supplement: S2 Table — (DOCX) [file pone.0288558.s002.docx]

## **S2 Table. Distribution of the answers given to the 5-point Likert scale evaluation questions halfway of the formative exam after MCQs (MCQfirst) or VSAQs (VSAQfirst).**

|  | **Regulation and Metabolism** | | | | | | **Diseases of the Abdomen** | | | | | |
| --- | --- | --- | --- | --- | --- | --- | --- | --- | --- | --- | --- | --- |
|  | **MCQ*first* (*n =* 104)** | | | **VSAQ*first* (*n* = 112)** | | | **MCQ*first* (*n* = 85)** | | | **VSAQ*first* (*n* = 64)** | | |
|  | EQ1 | EQ2 | EQ3 | EQ1 | EQ2 | EQ3 | EQ1 | EQ2 | EQ3 | EQ1 | EQ2 | EQ3 |
| 1: Strongly disagree | 1% | 12% | 0% | 2% | 12% | 2% | 2% | 0% | 0% | 0% | 15% | 2% |
| 2: Disagree | 10% | 46% | 10% | 15% | 45% | 4% | 5% | 38% | 10% | 5% | 61% | 7% |
| 3: Neutral | 32% | 39% | 13% | 28% | 39% | 8% | 19% | 58% | 36% | 15% | 25% | 10% |
| 4: Agree | 51% | 2% | 54% | 52% | 2% | 51% | 60% | 4% | 47% | 74% | 0% | 46% |
| 5: Strongly agree | 7% | 0% | 23% | 4% | 2% | 35% | 14% | 0% | 7% | 7% | 0% | 36% |

MCQ, multiple choice question; VSAQ, very short answer question.

EQ1: *The questions are a good representation of how I would be expected to answer questions in clinical practice.*

EQ2: *I found the questions easy.*

EQ3: *I was often unsure whether my answer would be correct.*
