# Supplementary material for: Use of very short answer questions compared to multiple choice questions in undergraduate medical students: An external validation study
Source: PLoS One. 2023 Jul 14;18(7):e0288558. doi: 10.1371/journal.pone.0288558 (PMC10348524; doi:10.1371/journal.pone.0288558)
Supplement: S3 Table — (DOCX) [file pone.0288558.s003.docx]

## **S3 Table. Distribution of the answers given to the 5-point Likert scale evaluation questions at the end of the formative exam.**

|  | **Regulation and Metabolism (*n =* 216)** | | | | | **Diseases of the Abdomen (*n* = 146)** | | | | |
| --- | --- | --- | --- | --- | --- | --- | --- | --- | --- | --- |
|  | EQ5 | EQ6 | EQ7 | EQ8 | EQ9 | EQ5 | EQ6 | EQ7 | EQ8 | EQ9 |
| 1: Strongly disagree | 59% | 9% | 8% | 9% | 24% | 63% | 4% | 9% | 4% | 12% |
| 2: Disagree | 31% | 21% | 22% | 24% | 44% | 29% | 13% | 21% | 19% | 36% |
| 3: Neutral | 7% | 36% | 19% | 42% | 26% | 3% | 23% | 24% | 41% | 37% |
| 4: Agree | 2% | 31% | 41% | 25% | 6% | 4% | 56% | 37% | 34% | 14% |
| 5: Strongly Agree | 0% | 3% | 10% | 1% | 0% | 0% | 4% | 9% | 2% | 1% |

EQ5: *VSAQs are easier than MCQs*.

EQ6: *VSAQs are more in line with daily clinical practice than MCQs.*

EQ7: *I prepare differently for an assessment with VSAQs than for an assessment with MCQs.*

EQ8: *VSAQs would be a better preparation for clinical practice than MCQs*.

EQ9: *Through the use of VSAQs, the test is better aligned with this course, than a test using MCQs.*
