# Supplementary material for: Use of very short answer questions compared to multiple choice questions in undergraduate medical students: An external validation study
Source: PLoS One. 2023 Jul 14;18(7):e0288558. doi: 10.1371/journal.pone.0288558 (PMC10348524; doi:10.1371/journal.pone.0288558)
Supplement: S4 Table — (DOCX) [file pone.0288558.s004.docx]

## **S4 Table. Median (IQR) scores and distribution of the answers given to the 5-point Likert scale evaluation questions after the summative exam.**

|  | **Regulation and Metabolism** | | **Diseases of the Abdomen** | |
| --- | --- | --- | --- | --- |
|  | **Q1 (*n* = 147)** | **Q2 (*n* = 148)** | **Q1 (*n* = 85)** | **Q2 (*n* = 85)** |
| 1: Strongly disagree | 13% | 53% | 26% | 18% |
| 2: Disagree | 26% | 30% | 27% | 33% |
| 3: Neutral | 17% | 12% | 6% | 19% |
| 4: Agree | 35% | 5% | 38% | 26% |
| 5: Strongly Agree | 10% | 0% | 4% | 5% |
| Median score (IQR) | 2 (3-4) | 1 (1-2) | 1 (2-4) | 2 (2-4) |

IQR, interquartile range.

Q1*: Because I knew that I would be tested by VSAQs, I studied in another way than I normally would;* Q2: *Through the use of VSAQs, the test was a better representation of what I learned in this course, than a test using MCQs.*
