# Supplementary material for: Use of very short answer questions compared to multiple choice questions in undergraduate medical students: An external validation study
Source: PLoS One. 2023 Jul 14;18(7):e0288558. doi: 10.1371/journal.pone.0288558 (PMC10348524; doi:10.1371/journal.pone.0288558)
Supplement: S5 Table — (DOCX) [file pone.0288558.s005.docx]

## **S5 Table**. **Median (IQR) scores of the 5-point Likert scale questions on constructive alignment after the summative exam (1: strongly disagree, 2: disagree, 3: neutral, 4: agree, 5: strongly agree).**

|  | **Regulation and Metabolism** | | | | | **Diseases of the Abdomen** | | | | |
| --- | --- | --- | --- | --- | --- | --- | --- | --- | --- | --- |
| **Q1** | | | **Q2** | | | **Q1** | | | **Q2** | |
|  | *N* | Median (IQR) | | *N* | Median (IQR) | *N* | Median (IQR) | *N* | | Median (IQR) |
| ‘16/’17 | *NA* | *NA* | | 197 | 4 (3-4) | *NA* | *NA* | 170 | | 4 (3-4) |
| ‘17/’18 | 50 | 3 (2-4) | | 50 | 4 (2-4) | 66 | 4 (3-4) | 66 | | 4 (3-4) |
| ‘18/’19 | 63 | 3 (2-4) | | 62 | 3 (2-4) | 62 | 2 (1-3) | 62 | | 2 (1-3) |
| ‘20/’21 | 149 | 2 (1-3) | | 149 | 2 (1-3) | 127 | 3 (2-4) | 127 | | 3 (2-4) |

IQR, interquartile range.

Q1: *The assessment as a whole (form and content) is appropriate for what you should have mastered at the end of the course.*

Q2: *The (online) test formats matched what I have learned; NA = not available.*
